# A small protein from the *bop–brp* intergenic region of *Halobacterium salinarum* contains a zinc finger motif and regulates *bop* and *crtB1* transcription

**DOI:** 10.1111/j.1365-2958.2007.06081.x

**Published:** 2008-02

**Authors:** Valery Y Tarasov, Hüseyin Besir, Rita Schwaiger, Kathrin Klee, Katarina Furtwängler, Friedhelm Pfeiffer, Dieter Oesterhelt

**Affiliations:** Max-Planck Institute of Biochemistry, Department of Membrane Biochemistry Am Klopferspitz 18, 82152 Martinsried, Germany

## Abstract

Bacteriorhodopsin, the photosynthetic protein of *Halobacterium salinarum*, is optimally expressed under anaerobic growth conditions. We identified Brz (OE3104F, bacteriorhodopsin-regulating zinc finger protein), a new regulator of the *bop* gene. It is a small protein with a zinc finger motif, encoded directly upstream of the *bop* gene in the same orientation. Deletion of the *brz* gene caused a large decrease of *bop* mRNA levels as shown by Northern blot and microarray analysis. A similar effect was obtained by site-directed mutagenesis of Cys and His residues in the zinc finger motif, indicating the importance of this motif for the function of the protein. *In silico* analysis of the genomes from *H. salinarum* and other archaea revealed a large family of similar small zinc finger motif proteins, some of which may also be involved in transcription regulation of their adjacent genes.

## Introduction

**Fig. 4:**
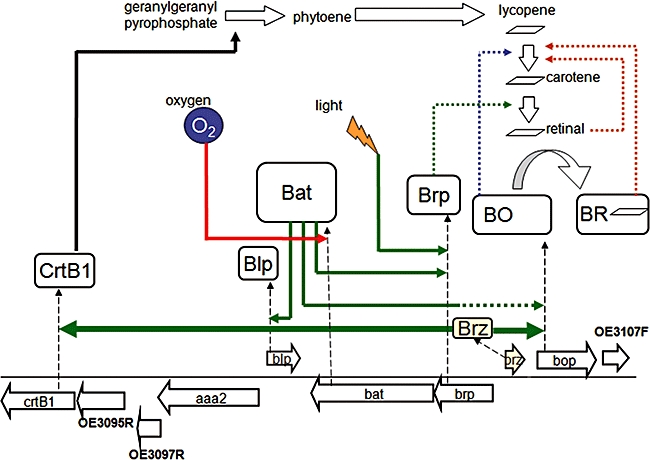
The *bop* gene regulation network. The *bop* gene cluster is displayed from the *crtB1* gene to a gene coding for a conserved hypothetical protein that follows the *bop* gene (OE3107F). The newly introduced *brz* gene (yellow) occurs in the region previously considered the intergenic region between *bop* and its assumed neighbour *brp*. Gene expression to the corresponding proteins is indicated by a dashed line. The proteins are indicated by boxes with corresponding capitalized gene names. For bacteriorhodopsin, the conversion of the apoprotein (BO, bacterio-opsin) to the mature protein (BR, bacteriorhodopsin) containing retinal (rhomboid) is indicated. Regulation of gene expression is indicated by green (induction) and red (inhibition) arrows. Expression of the *bat* gene is inhibited by oxygen while expression of the *brp* gene is enhanced by light (Shand and Betlach, 1991). The Bat protein is assumed to activate several genes (Shand and Betlach, 1991). The Bat protein activates *brp* gene expression (Leong *et al*., 1988a), *bop* gene expression (Leong *et al*., 1988b; Gropp and Betlach, 1994) and likely *blp* gene expression (Gropp *et al*., 1994). The Brz protein activates the *bop* and *crtB1* genes as shown in this manuscript (thick green arrows), but does not affect *bat* gene expression. This may indicate that the *bop* activation by Bat is not direct but mediated by Brz (green arrow broken beyond Brz). Also indicated is the conversion of geranylgeranyl pyrophosphate, phytoene and lycopene via beta-carotene to retinal. A negative feedback loop exists for which there are three alternative possibilities: substrate inhibition by retinal, inhibition by the bacterio-opsin apoprotein (brown dotted arrows) or activation by mature bacteriorhodopsin (blue dotted arrow) (Sumper and Herrman, 1976). Also indicated is the activation of carotenoid conversion to retinal by Brp (green dotted arrow) (Peck *et al*., 2001) and conversion of geranylgeranyl pyrophosphate to phytoene which is catalysed by *crtB1* (Baliga *et al*., 2001) (black arrow).

Bacteriorhodopsin (OE3106F, VNG1467G) is the key component of the retinal-based photosynthetic system of *Halobacterium salinarum*. It is the only protein in the purple membrane, forming two-dimensional crystals providing a means for photosynthetic growth under conditions of low-oxygen tension. The apoprotein bacterioopsin is encoded by the *bop* gene and covalently linked to retinal. The next gene upstream of *bop* is reported to be *brp* (*b*acterioopsin-*r*elated *p*rotein) (OE3102R, VNG1465G). It has been demonstrated that the integrity of the intergenic region between the *brp* and *bop* genes is important for *bop* expression. Insertions of ISH2 elements in this region (mutants W1 and W11) led to the inactivation of transcription of the *bop* gene (Pfeifer *et al*., 1985; Leong *et al*., 1988a). Insertions into *brp* also significantly decrease *bat* (*b*acterioopsin *a*ctivator of *t*ranscription) (OE3101R, VNG1464G) and *bop* mRNA levels (Pfeifer *et al*., 1985; Leong *et al*., 1988a). The effect on *bop* mRNA may result from a polar effect on the downstream gene *bat*, which forms a transcription unit with *brp* (Leong *et al*., 1988b; Shand and Betlach, 1991). In wild-type cells, both *bop* and *bat* transcription are induced during stationary phase (Yang and DasSarma, 1990; Shand and Betlach, 1991). Betlach and coworkers demonstrated that the *bat* gene encodes a *trans*-acting factor that induces *bop* at low-oxygen tension (Gropp and Betlach, 1994), which naturally occurs in stationary phase. Bat contains a GAF domain, a PAS/PAC (redox-sensing) domain, and a C-terminal DNA-binding helix–turn–helix motif (Baliga *et al*., 2001). *brp* and a second gene *blh*, located 500 kb from the *bop* locus, have been implicated in retinal synthesis as an in-frame *brp* deletion led to the accumulation of β-carotene and a decrease of retinal (Peck *et al*., 2001). Adjacent to *bat* is *blp* (*b*op-*l*inked *p*rotein) (OE3100F, VNG1463G), which is co-regulated with the *bop* gene by low-oxygen tension (Gropp *et al*., 1994) and encodes a protein with unknown function. Three genes upstream of *blp* are the *crtB1* gene (OE3093R, VNG1458G) encoding phytoene synthase, the key enzyme in the biosynthesis of C_40_ carotenoids, and thus retinal biosynthesis (Baliga *et al*., 2001) (see Fig. 4 for a scheme illustrating regulation of bacteriorhodopsin synthesis).

The genomes of two strains of *H. salinarum* have been sequenced (Ng *et al*., 2000; Pfeiffer *et al*., 2007; http://www.halolex.mpg.de), and they were found to be nearly identical in their chromosomal sequences. An exceedingly small open reading frame (ORF) (44 residues) (OE3104F, VNG1466H) was found annotated in the intergenic region between *bop* and *brp*, which was considered one of the many spurious ORFs that are characteristic for this GC-rich (68% GC) genome.

In this study, we show that the small protein, after correction of the start codon assignment, contains a zinc finger motif. The requirement of the protein and its proposed zinc finger for efficient *bop* gene transcription was shown by gene deletion, site-directed mutagenesis and microarray analysis. We propose the name *brz* (bacteriorhodopsin-regulating zinc finger protein) for this gene. In addition, we show that a family of similar small proteins with a zinc finger motif is found not only in the genome of *H. salinarum*, but also in all archaea.

## Results

### The gene upstream of *bop* is *brz* and not *brp*

**Fig. 1:**
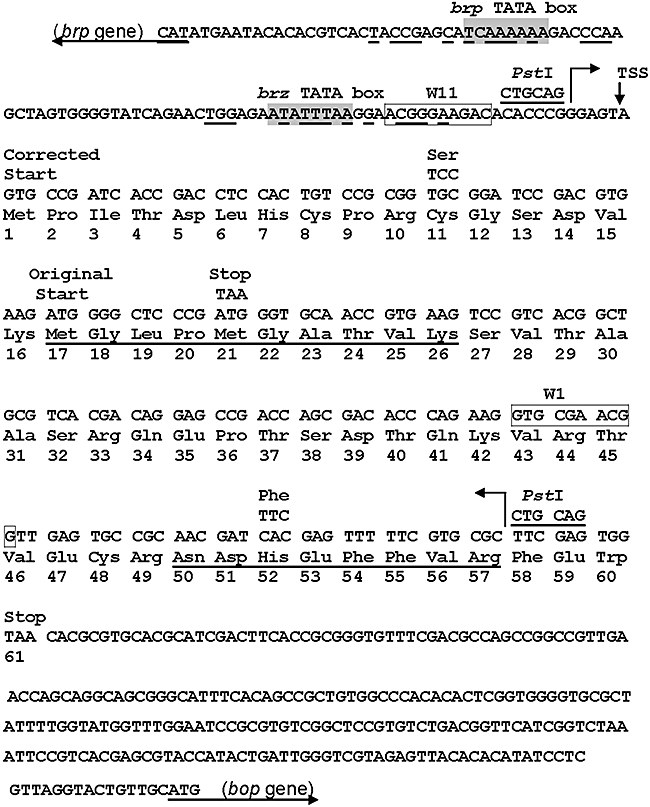
Position of the *brz* gene in the intergenic region between the *brp* and *bop* genes. The sequence of the Brz protein is depicted below the gene. The peptides identified in the proteome analysis are underlined. Numbers indicate the positions of amino acid residues in Brz after correction of the start codon. The positions of the corrected and original start codons are indicated. TSS is the transcription start site determined by 5′ RACE. The TATA boxes of the *brz* and *brp* genes are indicated by grey boxes. Nucleotides forming an imperfect inverted repeat in the putative promoter regions are underlined. The target duplications at the ISH2 element integration sites of mutants W11 and W1 are boxed (Pfeifer *et al*., 1985; Leong *et al*., 1988a). The sequence modifications of four mutants are also shown: (i) The bent arrows indicate the extent of the deletion mutant (Δ*Brz*). Artificial PstI restriction sites were introduced at the start and end of the deletion. (ii) In mutant *stopBrz*, the ATG for Met-21 was converted to a stop codon (indicated as Stop above the sequence). (iii, iv) Nucleotide and corresponding amino acid substitutions made in mutants *BrzC11S* and *BrzH52F* are indicated above the corresponding codons.

The *bop* and *brp* genes with their intergenic region had been sequenced (Dunn *et al*., 1981; Betlach *et al*., 1984) and, ever since it was assumed that *brp* is the gene directly upstream of *bop*. On the genome sequence of *H. salinarum* (Ng *et al*., 2000; Pfeiffer *et al*., 2007; http://www.halolex.mpg.de), an ORF of only 44 residues in the intergenic region between *bop* and *brp* was annotated (OE3104F, VNG1466H). In light of the detailed analysis of the region upstream of *bop*, and considering that the GC-rich genome of *Halobacterium* contains ORFs of up to 1300 residues which do not code for a protein, it was considered unlikely that ORF OE3104F is a gene. This changed, when a protein translated from OE3104F was identified in our proteomic survey which was specifically tailored to study the small proteome (Klein *et al*., 2007). It was also realized that an alternative GUG start codon exists 16 codons upstream resulting in a 60-amino-acid protein (Fig. 1). Using 5′ RACE (rapid amplification of cDNA ends), we identified the transcription start site (TSS) to be the adenine preceding the GUG start codon (Fig. 1). The N-terminal extension of the protein contains two Cys residues and one His residue in addition to the His and Cys residues near the C-terminus of the protein. With the additional pair of Cys residues, the extended protein contains a zinc finger like motif, which is a well-known motif found in transcriptional and translational regulators, making it likely that this gene product is involved in gene regulation. Therefore, we named the gene *brz*. In addition, the 100 bp intergenic region between the *brp* and *brz* genes contains an imperfect inverted repeat which overlaps with the putative promoters of the two genes (Fig. 1, underlined). This repeat includes a putative TATA box of the *brz* promoter (located 25 bp upstream of the identified TSS) and a putative TATA box for *brp*. This suggests that *brz* and *brp* could be co-regulated and could ultimately be involved in the regulation of *bop* gene expression.

### *brz* is required for high *bop* and *crtB1* mRNA levels

The influence of the *brz* gene for the *bop* mRNA level was demonstrated by gene deletion and site-directed mutagenesis. We constructed four mutants: a deletion strain (*ΔBrz*) and a mutant (*stopBrz*) containing an in-frame stop codon replacing Met-21 [60 bp downstream of the corrected and 12 bp downstream of the original start codon (Fig. 1)]. The mutant *stopBrz* should not be able to produce any functional protein, regardless of which potential start codon is used by the organism. Further, we mutated both Cys-11 to Ser (BrzC11S) to clarify the role of the alternative amino terminal sequence in formation of the proposed zinc finger motif, and His-52 to Phe (BrzH52F) to check the other part of the zinc finger motif. All mutations were confirmed by sequence analysis (see *Experimental procedures* and *Supplementary material*).

**Fig. 2:**
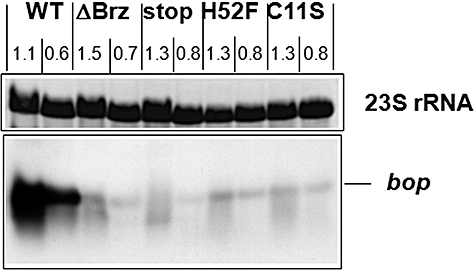
Effects of four *brz* mutations on the mRNA level of *bop*. The upper block represents the 23S rRNA bands on the agarose gel stained by ethidium bromide. The lower block shows a Northern blot of total cell RNA probed with DNA fragments containing the *bop* gene. Numbers indicate OD_600_ of cell cultures used for RNA preparation (0.6–0.8 exponential phase; 1.1–1.5 stationary phase). WT, wild-type strain R1; Δ*Brz*, *brz*-deletion strain; Stop, strain *stopBrz*; H52F and C11S, strains *BrzH52F* and *BrzC11S*.

In all mutants, the level of *bop* mRNA was dramatically reduced in comparison with wild-type level as shown by Northern blot data (Fig. 2). The effects in the point mutants, BrzC11S and BrzH52F, were as extensive as those in the deletion mutant, indicating involvement of the zinc-finger-like motif in the function of Brz. The analysis was performed at two cell densities: 0.6–0.8 OD_600_ (late exponential phase) and 1.1–1.5 OD_600_ (early stationary phase) as *bop* transcription is induced at stationary phase.

To identify additional targets for Brz and check for an effect on genes of the *bop* regulon, we carried out whole-genome microarray experiments comparing transcriptional profiles of the *brz* mutants with wild-type strain R1. The list of down- and upregulated genes for the deletion mutant is presented in Table 1, that for the other mutants in Table S1. The decrease of mRNA levels of the *bop* gene could be confirmed for all mutants. In all four mutants, we found reduced mRNA levels for the *crtB1* gene (VNG1458G, OE3093R), which encodes phytoene synthase, catalysing an early step of retinal biosynthesis. Two other genes from the immediate vicinity of the *bop* gene are downregulated. The gene for OE3107F is located directly downstream of the *bop* gene, not only in *H. salinarum* but also in *Haloquadratum walsbyi* and *Haloarcula marismortui*. The conserved gene pairing in the three bacteriorhodopsin-containing halophiles may indicate functional association. OE3107F is a protein with two predicted transmembrane domains. The gene for OE3095R is located directly upstream of the *crtB1* gene. Orthologous genes are located directly upstream of the *bop* gene in *H. walsbyi* and *H. marismortui*. Protein OE3095R, which is 133 residues long, also contains a zinc finger motif similar to that of Brz.

**Table 1:** Downregulated and upregulated genes as well as *bop*-related non-regulated genes in a response to deletion of the *brz* gene of *H. salinarum*

ID	Regulation factor	Gene	Protein name
Downregulated genes in the *ΔBrz* mutant			
OE3095R	−5.3	–	Hypothetical protein
OE3093R	−3.1	*crtB1*	Phytoene synthase
OE3107F	−2.8	–	Conserved hypothetical protein
OE3106F	−2.5	*bop*	Bacteriorhodopsin precursor
OE4427R	−2.3	*dpsA*	Ferritin
OE4670F	−2.3	–	Conserved hypothetical protein
Upregulated genes in the *ΔBrz* mutant			
OE6130F	9.7	–	Conserved hypothetical protein
OE6098R	5.5	–	Conserved hypothetical protein
OE2100R	5.0	*spoVR*	Spore cortex formation protein homologue
OE6157R	4.9	–	Hypothetical protein
OE2442R	3.0	–	Hypothetical protein
OE3766R	2.9	–	Hypothetical protein
OE6097R	2.7	–	Conserved hypothetical protein
OE4313F	2.4	*appB*	ABC-type transport system permease protein
OE4311F	2.2	*appA*	ABC-type transport system periplasmic substrate-binding protein
OE6099F	2.1	–	Hypothetical protein
OE1409F	2.1	–	Conserved hypothetical protein
OE6096A1R	2.0	–	Hypothetical protein
OE2906R	*2.0*	*sod2*	Superoxide dismutase 2
OE7194F	*2.0*	*repJ1*	Plasmid replication protein repJ
Non-regulated *bop*-related genes in the *ΔBrz* mutant			
OE3102R	1.1	*brp*	Bacteriorhodopsin-related protein
OE3100F	−1.6	*blp*	Bacterioopsin-linked protein blp
OE3101R	1.0	*bat*	Bacterioopsin activator
OE2448F	1.2	*boa4*	Homologue to transcription regulator bat
OE3134F	[1.0]	*boa2*	Homologue to transcription regulator bat
OE3980R	−1.1	*blh*	Brp-like protein

Regulation factors represent the relative intensity as computed from the log_2_ ratio. Negative values indicate downregulation while positive values indicate upregulation. The term ‘regulation factor’ is also used for the *bop*-related but non-regulated genes. Values in square brackets indicate data with a false discovery rate above 5% which is a consequence of the differences being minimal (‘regulation’ factors below 1.2).

The *bat* gene, encoding a known regulator of *bop* transcription, revealed unchanged mRNA levels in the mutants (Table 1, Table S1). Northern blot analysis confirms the microarray results for the *bat* gene (data not shown). This excludes an indirect effect of Brz on *bop* transcription via the direct deregulation of the *bat* gene. Among the other genes from the *bop* regulon which do not show alterations in transcription level are *brp*, *blp*, *boa2*, *boa4* and *blh* (Table 1, Table S1).

### Brz belongs to a large family of small zinc finger proteins in *H. salinarum*, archaea and bacteria

**Fig. 3:**
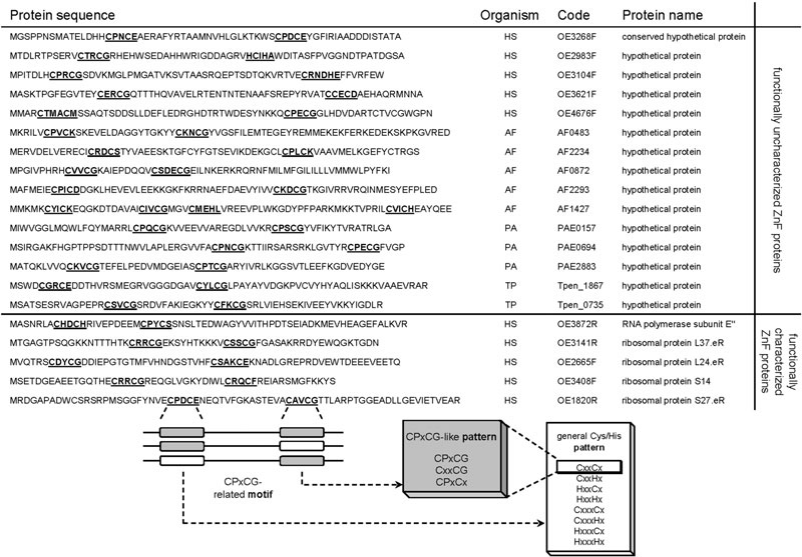
A selection of proteins containing a CPxCG-related zinc finger motif and definition of patterns and motifs to detect them. A large set of proteins shorter than 100 amino acids contain a CPxCG-related zinc finger motif. Several examples from *H. salinarum* (HS), *Archaeoglobus fulgidus* (AF), *Pyrobaculum aerophilum* (PA) and *Thermofilum pendens* (TP) are listed. The upper part shows hypothetical and conserved hypothetical proteins which are unrelated to each of them outside the zinc finger patterns. The proteins in the lower part are functionally characterized. The zinc finger patterns are indicated by bold underlining. A definition of patterns and motifs is provided below the sequences. A CPxCG-related zinc finger motif consists of a pair of patterns, separated by 7–40 amino acids. One of the patterns must be a CPxCG-like pattern (grey), of which three forms exist. The other can be a more general Cys/His pattern containing two Cys or His residues separated by two or three intermediate amino acids (white). All eight types of the general Cys/His pattern are shown, the CPxCG-like pattern being a specification of one of them (CxxCx).

Proteomic experiments for *H. salinarum* identified many other small proteins with a zinc finger motif in addition to Brz (Klein *et al*., 2007). *In silico* analysis allowed a more specific motif definition, which we refer to as ‘CPxCG-related zinc finger motif’ (Fig. 3, for details see *Experimental procedures*). It consists of two patterns which are 7–40 residues apart as revealed by distance analysis. Each pattern is based on a general Cys/His pattern (two Cys or His residues separated by two to three intermediate amino acids), and may have the specific form of a CPxCG-like pattern (CPxCG, CPxCx, CxxCG). At least one CPxCG-like pattern is required for a CPxCG-related zinc finger motif.

The majority of the proteins containing this motif are annotated as ‘(conserved) hypothetical protein’ (for example sequences see Fig. 3), but also a number of proteins involved in DNA and RNA interaction have been found. Among those are ribosomal proteins, small subunits of RNA polymerase, and transcription initiation and translation factors. The majority of the proteins detected by our algorithm did not show any hit to the Prosite motif database. However, there was a moderate cross-identification of iron–sulphur proteins (4Fe−4S ferredoxin, rubredoxin), which could be diminished by motif-based filtering.

One-fourth of the proteins having a CPxCG-related zinc finger motif are shorter than 100 residues (less than 12 kDa). The frequency maximum is at 50–70 residues as revealed by statistical analysis of the annotated proteins from 32 completely sequenced archaeal genomes. Among the very small archaeal proteins, a remarkably large fraction (8%) has a CPxCG-related zinc finger motif, and thus may interact with DNA or RNA (Table S4). Even this may be an underestimation according to our *in silico* analysis. A search for yet unannotated short six-frame translation products with a CPxCG-related zinc finger motif indicates that many of these may have been overlooked so far.

Upon analysis of 24 bacterial genomes, selected to represent a broad phylogenetic spectrum, a number of small proteins with a CPxCG-related zinc finger motif were also found (Table S5). However, these were not as frequent as in archaea. Only 1.5% of the annotated proteins smaller than 100 amino acids contained this motif, although some additional candidates were found by six-frame translation analysis. As an example, there are only 12 small zinc finger proteins (3%) in *Escherichia coli* and eight (1.7%) in *Bacillus subtilis*. A distinct exception is the genome of *Salinibacter ruber* which contains many archaeal traits (Mongodin *et al*., 2005). This species contains 20 (11%) small zinc finger proteins.

Overall, our bioinformatic results point to the existence of a prominent class of small proteins which may regulate gene expression by interaction with DNA or RNA.

## Discussion

The newly characterized gene between the *brp* and *bop* genes codes for a small zinc finger protein which regulates *bop* gene transcription and was named *brz*. Upon deletion of the gene or its inactivation by an in-frame stop codon, transcription of the *bop* gene is strongly impaired as shown by Northern blot (Fig. 2) and microarray analysis (Table 1, Table S1). While the effects detected by Northern analysis were very strong (Fig. 2), results from DNA microarray analysis show only moderate regulation factors for *bop*. This may be due to a low dynamic range of DNA microarray analysis [as has also been found in other studies with this organism (Twellmeyer *et al*., 2007)]. The same technique was used to confirm that the CPxCG-related zinc finger motif has an important role in the function of the protein. Site-directed mutagenesis of a Cys residue from the N-terminal pattern (C11S), as well as a His residue from the C-terminal pattern (H52F), also strongly impaired *bop* gene transcription. The C11S mutant also supports re-annotation of the start codon for the *brz* gene.

Our data allow a reinterpretation of earlier observations showing that insertion of ISH2 elements in the ‘intergenic region’ between *brp* and *bop* leads to the inactivation of *bop* transcription (Pfeifer *et al*., 1985; Leong *et al*., 1988a). The integration sites of the ISH2 elements are located within the *brz* gene (strain W1) or only 13 bp upstream of the newly assigned *brz* start codon (strain W11) (Fig. 1). Thus, both integration events directly affect the *brz* gene rather than occurring in an intergenic region.

Brz functions within the overall context of *bop* gene regulation which involves several gene products, most of which are encoded in the immediate vicinity of the *bop* gene (*bop* cluster) (Fig. 4). As the product of the *bat* gene is known to regulate *bop* transcription (Leong *et al*., 1988b; Gropp *et al*., 1994), we analysed the effects of the different *brz* mutations on *bat* transcription by Northern blot (data not shown) and microarray (Table 1, Table S1). While mutation of *brz* had a major effect on *bop* transcription, it did not affect *bat* transcription. The same effects were observed earlier in the W1 and W11 strains having ISH2 element insertions (Leong *et al*., 1988a). Accordingly, the regulatory effects of Brz on *bop* are not mediated via Bat. The opposite scenario that Bat exerts its effects via regulation of *brz* cannot be excluded. Another possibility is that Bat and Brz cooperate on the protein level for *bop* gene regulation. Such an interaction is indirectly supported as the two Bat homologues of *H. salinarum* are both accompanied by genes coding for additional small zinc finger proteins with a CPxCG-related zinc finger motif. Adjacent to the *bat* homologue *boa4* (OE2448F) is the gene for the small zinc finger protein OE2447F. The *bat* homologue *boa2* (OE3134F) is separated by a single gene from the gene for the small zinc finger protein OE3131F. Regulation of the *brz* gene itself is likely and is supported by the imperfect inverted repeat observed in the intergenic region between *brz* and *brp* which overlaps the promoter region of both genes.

We performed DNA microarray analysis with the four *brz* mutants also in order to identify additional targets. In addition to the *bop* gene, three genes consistently showed reduced transcript levels for all four *brz* mutants, and they are all located in the immediate vicinity of the *bop* gene. Among those is a second key target involved in bacteriorhodopsin biosynthesis, the *crtB1* gene (OE3093R). It encodes the phytoene synthase catalysing the first step of carotenoid biosynthesis. Beta-carotene is the immediate precursor of the bacteriorhodopsin chromophore retinal. Thus, *brz* is a new member of those regulators which affect bacteriorhodopsin production at the protein level (*bop* gene regulation) and on the pigment level (*crtB1* gene regulation) (Fig. 4). Co-regulation of *bop* and *crtB1* at the transcription level may occur by binding to the upstream activator sequence of the two promoters (Baliga and DasSarma, 1999), for which sequence similarities have been reported (Baliga *et al*., 2001). Alternatively, *crtB1* regulation may be an indirect effect mediated via *bop* regulation. The indirect regulation of *bop* via *crtB1* can be excluded, as this would require accumulation of free retinal, which is never observed.

The other two genes which show reduced transcript levels upon DNA microarray analysis of all four *brz* mutants are: (i) *OE3107F*, the gene directly downstream of the *bop* gene and (ii) *OE3095R*, the gene immediately upstream of the *crtB1* gene. In both cases, however, gene distances are large enough to support independent transcription. Interestingly, these two genes are the direct neighbours of *bop* genes in two other bacteriorhodopsin-containing halophiles, *H. walsbyi* and *H. marismortui*. As for *H. salinarum*, the OE3107F homologues are encoded directly downstream of a *bop* gene. The homologues to OE3095R are encoded directly upstream of the same *bop* gene in opposite orientation. This resembles relative gene positions of *H. salinarum* except that a cassette containing six genes, among them *brz*, *brp*, *bat* and *blp*, has been inserted before the *bop* gene. These two co-regulated genes are not found in the closely related halophile *Natronomonas pharaonis* which does not contain bacteriorhodopsin, although it contains other retinal proteins (halorhodopsin, sensory rhodopsin II). OE3095R, which is 133 residues long, also contains a CPxCG-related zinc finger motif. This opens the possibility for a hierarchical regulatory network consisting of more than one zinc finger protein. Interaction between different gene regulators having a CPxCG-related motif may also be responsible for the fact that a number of additional genes were found to have reduced (or increased) mRNA levels, but only for some of the four mutants. The affected genes may even show a more prominent regulation factor than bacteriorhodopsin in DNA microarray analysis.

*In silico* genome analysis revealed a new large class of small proteins possessing a CPxCG-related zinc finger motif similar to that in Brz. In our analysis, we concentrated on very short proteins (below 100 residues, i.e. below 12 kDa) which most likely are devoid of additional structural domains. Such small proteins are notoriously difficult to deal with. As we have recently shown (Klein *et al*., 2007), small proteins have been systematically overlooked because of technical problems related to gel electrophoresis (protein washout) and proteomic analysis (low peptide numbers). Our data point to the high relevance of small zinc finger proteins in archaea and, to a lesser extent, in bacteria. A remarkably large fraction of the small proteins in 32 completely sequenced archaea contains a CPxCG-related zinc finger motif. On average, 8% of the small proteins contain such a motif (one in 12 proteins). This may even be an underestimation, as additional candidates were detected in yet unannotated six-frame translations. The proteins are also found in bacteria, but to a much lesser extent, as shown by analysis of 24 bacterial genomes. On average, 1.5% of the proteins contain a CPxCG-related zinc finger motif. A notable exception is *S. ruber*, which does not only contain a high percentage of CPxCG-related zinc finger motifs, but is otherwise also reported to have an extensive set of probably archaea-derived genes (Mongodin *et al*., 2005).

If the small proteins with a CPxCG-related zinc finger protein are gene regulators in a way similar to what we have shown for Brz, then a new chapter of gene regulation analysis in archaea has to be opened. The current report is thus only a starting point. Further experiments are ongoing to analyse the role of the other small CPxCG-related zinc finger proteins with respect to functional specificity, affected targets and the general function mechanism.

## Experimental procedures

### Strains and growth conditions

*Halobacterium salinarum* R1 and mutant strains derived from R1 were grown as described (Cline and Doolittle, 1987). The *E. coli* strain XL1-Blue was used for transformation which was carried out according to Inoue *et al*. (1990).

### Construction of the pVT11, pVT12, pVT13, pVT14 mutagenesis vectors and *ΔBrz, stopBrz, BrzC11S, BrzH52F* mutants of *H. salinarum*

The pVT plasmid was obtained by cloning the blunted HindIII–XbaI fragment containing the *bgaH* gene from the pMLH32 plasmid (Holmes and Dyall-Smith, 2000) into the SmaI site of the pAN plasmid (Tarasov *et al*., 2000). The *bgaH* gene and NovR are oriented in opposite directions in pVT. The pVT11, pVT12, pVT13, pVT14 plasmids were obtained by cloning Δ*Brz*, *stopBrz*, *BrzC11S* and *BrzH52F* fragments into the pVT plasmid using HindII, BamHI, XbaI restriction sites (generation of Δ*Brz*, *stopBrz*, *BrzC11S*, *BrzH52F* fragments is described in *Supplementary material*: PCR amplification and construction of the Δ*Brz*, *stopBrz*, *BrzC11S*, *BrzH52F* fragments). The fragments were verified by sequencing of the corresponding plasmids in both directions using the universal M13/pUC reverse primer and the reverse primers designed for PCR amplification (Table S2). The pVT11–pVT14 plasmids do not contain a haloarchaeal origin of replication and, after transformation, they integrate into the chromosome by recombination. Transformations were carried out by the PEG method with modifications as described (Cline *et al*., 1989; Tarasov *et al*., 2000). Transformants were selected using blue/red screening (Koch and Oesterhelt, 2005), by plating the cells onto agar growth medium containing 0.1–0.2 μg ml^−1^ novobiocin (Sigma, USA) and 40 μg ml^−1^ Xgal (Patenge *et al*., 2000). Single blue colonies were picked and propagated in culture medium without novobiocin to allow a second cross-over event. Diluted cells were then plated on agar plates containing 40 μg ml^−1^ Xgal without novobiocin, and red colonies were checked for the presence of the respective mutations by sequencing of PCR fragments. For the amplification and sequencing of these fragments, the fp1, rp1 primers were used (Table S2).

### Northern blot hybridizations and 5′ RACE

Northern blot hybridizations were done as described (Tarasov *et al*., 2000). Digoxigenin-labelled *bop* and *bat* gene probes generated by PCR were used for the chemiluminescence detection performed with the DIG luminescence detection kit (Roche) according to the supplier's instructions. The following primer pairs were used: fp6–rp6 and fp7–rp7, respectively, for the *bop* and *bat* gene PCR amplification (Table S2). Total RNA was prepared using the peqGold RNAPure kit (Peqlab Biotechnology) according to the supplier's instruction.

The 5′ RACE was performed using the 5′ RACE System, Version 2.0 (Invitrogen). First strand cDNA was generated from 3 μg of total RNA using the GSP1 primer (5′-GCGGCACTCAACCGTTCGCACC-3′). RACE-PCR was carried out by using the tailed cDNA as template and GSP2 (5′-GCTGGTCGGCTCCTGTCGTGA-3′) and AAP (Invitrogen) primers according to the supplied protocol (Invitrogen). The obtained PCR-amplified fragments were cloned (TOPO TA cloning Kit, Invitrogen) and sequenced using T3 and T7 oligonucleotides.

### Microarray analysis

RNA was prepared using the peqGold RNAPure kit (Peqlab Biotechnology), and the contaminating DNA was digested with DNase I following the manufacturer's instructions (Ambion). RNA quality was checked with the 2100 Bioanalyzer (Agilent). RNA was transcribed into Cy3/Cy5-labelled cDNA (CyScribe First-Strand cDNA Synthesis Kit) using random nonamer primers (Amersham Biosciences). Afterwards, the reaction was stopped, the RNA template chemically degraded and the cDNA cleaned and concentrated (for detailed instructions see Zaigler *et al*., 2003). The cleaned cDNA was hybridized to microarrays according to Zaigler *et al*. (2003). Microarrays were manufactured according to Diehl *et al*. (2001). Five replicate probes of cleaned PCR products for each gene were spotted on GAPSII glass slides (Corning). The fluorescence images of microarrays were made by using the scanner 4000B (Axon). The data extraction was done by GenePix Pro 6 software (*Supplementary material*).

For comparison of the wild-type strain R1 with *brz* mutants, two microarrays were used. These technical replications were designed as dye-swap experiments. All microarray experiment data are deposited at EBI ArrayExpress, and are accessible under the accession number E-MEXP-1300 (MIAMExpress).

### Detection algorithm for small zinc finger proteins having a CPxCG-related zinc finger motif

The principle of the detection algorithm for the CPxCG-related zinc finger motif is described here, full details being available as *Supplementary material*. The algorithm is based on the identification of a CPxCG-like pattern (CPxCG, CxxCG, CPxCx) and a more general Cys/His pattern (two Cys or His separated by two to three intermediate amino acids). A CPxCG-related zinc finger motif consists of two paired patterns, of which at least one must be CPxCG-like. Based on initial data analysis, we allow pattern pairing only within the distance range of 7–40 residues. Motif analysis of the resulting protein set against the Prosite database (Hulo *et al*., 2006) revealed a considerable (*c*. 15.5%) contamination with iron–sulphur proteins (4Fe−4S ferredoxins, rubredoxins). Negative filters using the Prosite motifs PS00198 and PS00202 were implemented to reduce these contaminations. Many of the proteins thus identified are very short. A distinct frequency maximum in the protein size range of 50–70 residues was identified. We introduced a length cut-off of 100 amino acids (proteins thus being below 12 kDa). Further analysis is restricted to these ‘small zinc finger proteins’, i.e. proteins which have at least one CPxCG-related zinc finger motif and which are shorter than 100 residues. More details of the algorithm and the results of the initial analyses are detailed in the *Supplementary material*.

### Identification of small zinc finger proteins in archaeal and bacterial genomes

We identified small zinc finger proteins in 32 completely sequenced archaeal and several bacterial genomes. To allow detection of yet unannotated proteins, we used six-frame translation data with a size limit of 30 amino acids. Six-frame ORFs were mapped to the annotated protein-coding gene set based on the unambiguous position of the stop codon. This allows to distinguish between annotated and newly detected small zinc finger proteins. Full statistical data are specified in the *Supplementary material*.

## Supplementary material

This material is available as part of the online article from: http://www.blackwell-synergy.com/doi/abs/10.1111/j.1365-2958.2007.06081.x

(This link will take you to the article abstract).
