# Supplementary material for: A small protein from the bop–brp intergenic region of Halobacterium salinarum contains a zinc finger motif and regulates bop and crtB1 transcription
Source: Mol Microbiol. 2008 Feb;67(4):772–80. doi: 10.1111/j.1365-2958.2007.06081.x (PMC2253796; doi:10.1111/j.1365-2958.2007.06081.x)
Supplement: Supplementary file 1 [file mmi0067-0772-SD1.pdf]

Supplementary Material

PCR amplification and construction of the ΔBrz, stopBrz, BrzC11S, BrzH52F fragments

The *stopBrz*, *BrzC11S*, *BrzH52F* fragments were amplified by splicing PCR of pairs of fragments *stopBrz*(1), *BrzC11S*(1), *BrzH52F*(1) and *stopBrz*(2), *BrzC11S*(2), *BrzH52F*(2), accordingly, using primers: fp1, rp1 (Suplementary Table S2). The primers include restriction sites for cloning: fp1 - *Hind*III, rp1 – *Xba*I. The *stopBrz*(1), *BrzC11S*(1), *BrzH52F*(1) and *stopBrz*(2), *BrzC11S*(2), *BrzH52F*(2) fragments were previously PCR amplified from genomic DNA using the following oligonucleotide pairs: fp1-rp2, fp1-rp3, fp1-rp4 and fp2-rp1, fp3-rp1, fp4-rp1 respectively. The *ΔBrz* fragment was constructed by ligation of *ΔBrz*(1) and *ΔBrz*(2) after digestion with the restriction enzyme *Pst*I. *ΔBrz*(1) and *ΔBrz*(2) were amplified using the fp1, rp5 and fp5, rp1 primers (Suplementary Table S2).

Data analysis

The raw values were further processed in the R environment with programs written by G. Welzl (Twellmeyer *et al.*, 2007). Processing is based on local background substraction and blockwise median normalization. Unreliable low intensity values were identified by the minimum volume ellipsoid method. The datasets were evaluated using a Student's t-Test (Tusher *et al.*, 2001) as implemented in the TIGR Multiexperiment Viewer (Saeed *et al.*, 2003). Genes within a False Discovery Rate (FDR) of 5% and a $\log_2$ ratio of more/less than 1/-1 were selected as being significantly regulated. The standard deviation of $\log_2$ ratios of the 5 replicate spots on the microarray was smaller than the $\log_2$ ratio.

Detection algorithm for small zinc finger proteins having a CPxCG-related zinc finger motif

Analysis was initiated by detecting that Brz belongs to a larger set of small zinc finger proteins having a CPxCG-like sequence pattern. A zinc finger motif is formed by two

patterns, each having two closely spaced Cys or His residues. We consider patterns which consist of two Cys and/or His residues separated by 2 or 3 intermediate amino acids (referred to as general Cys/His pattern). This results in eight pattern types (CxxCx, CxxHx, HxxCx, HxxHx, CxxxCx, CxxxHx, HxxxCx, HxxxHx). The CPxCG-like pattern (CxxCG, CPxCx, CPxCG) is a special form of the more general pattern type CxxCx. A CPxCG-related zinc finger motif contsists of two Cys/His patterns of which at least one is CPxCG-like.

In a first analysis we searched for distance constraints in pairs of Cys/His patterns. The processing procedure is illustrated in Supplementary Fig. S1. Candidates were selected from the 74714 annotated ORFs from 32 completely sequenced archaeal genomes. A total of 4475 proteins fulfil the minimal criteria to have at least 2 Cys/His patterns of which at least one is CPxCG-like. This protein set was further restricted as to minimize ambiguities and therefore only the 2054 proteins with exactly two patterns were further considered. When requesting that both patterns were CPxCG-like, 864 proteins remained. Distance analysis for these allowed to define a length constraint of 7-40 residues (Supplementary Fig. S2) which covers 97% of the proteins in the set although no contstraint has been applied concerning total protein length. Pattern pairs with a distance less than 7 residues were not observed.

This analysis was repeated under more relaxed conditions where only one of the patterns was CPxCG-like, the other being a general Cys/His pattern. Also in this analysis, the length constraint of 7-40 residues applies as indicated by a clear plateau in the cumulative plot (Supplementary Fig. S3). However, only 70% of the proteins contain pattern pairs within this distance. The remaining 30% have much longer pattern distances and are not considered relevant in the context of the current analysis. Eight different forms of the Cys/His pattern have been included in our selection procedure. We determined the relative frequency for patterns which are not CPxCG-like. Pairs with a distance of 7-40 residues show a strong preference for the CxxCx type (Supplementary Fig. S4). In contrast, such a preference is not

found for long distance pattern pairs which supports our decision to exclude them from further analysis.

Based on these results, the detection algorithm was improved especially concerning pattern selection in very closely spaced or overlapping patterns (Supplementary Fig. S5). (i) ***Overlapping patterns***: Overlapping Cys/His patterns may occur when more than two Cys or His residues are closely spaced. For motifs having three intermediate residues, neither Cys nor His is allowed at the second position. This resolves ambigous patterns like CCxxCx such that the CxxCx pattern is extracted (Supplementary Fig. S5A), consistent with the strong over-representation of the shorter Cys/His pattern that was found before. Also, a CPxCG-like pattern is extracted from overlapping patterns if possible. Otherwise, the most leftward pattern is extracted (Supplementary Fig. S5B). (ii) ***Very closely spaced patterns***: Pattern distances below 7 residues were not observed when requesting that both pattern are CPxCG-like and thus such closely spaced patterns were specifically processed. If one of the patterns is CPxCG-like and the other is a general Cys/His pattern, then only the CPxCG-like pattern is extracted. If both patterns are CPxCG-like or both are general, then both patterns are extracted (Supplementary Fig. S5C). (iii) ***Distance-based pairing of patterns to CPxCG-related zinc finger motifs***: Only paired patterns which are 7-40 residues apart are defined to be zinc finger motifs. At least one of the pairs must be a CPxCG-like patterns.

Proteins were extracted by the improved algorithm and subjected to motif searches against the Prosite database (Hulo *et al.*, 2006) using the ps_scan program (Gattiker *et al.*, 2002). Analysis of 3674 proteins revealed a moderate (~15.5%) contamination with iron-sulfur proteins (4Fe-4S ferredoxins, rubredoxins). There were 553 occurences of motif PS00198 (“4FE4S_FERREDOXIN”), 17 (0.5%) occurences of motif PS00202 (“RUBREDOXIN”) and 57 (1.6%) occurences of matrix PS50903 (“RUBREDOXIN-LIKE”). Negative filters using the Prosite motifs PS00198 and PS00202 were implemented to reduce these contaminations. No other motif occurred with very high abundancy, indicating that the

CPxCG-related zinc finger motif described in this manuscript is not yet represented in the Prosite database. Several motifs from zinc finger proteins or other proteins interacting with DNA and RNA were moderately common but only 4 occurred more than 50 times (PS51134: "ZF_TFIIB", PS00782: "TFIIB", PS00178: "AA_TRNA_LIGASE_I", PS51192: "HELICASE_ATP_BIND_1"). Attempts to improve the specificity of the CPxCG-related zinc finger motif (analysis by two motif servers, computation of position-specific amino acid frequencies) were not successful.

Proteins were extracted by the final form of the algorithm and protein length statistics were computed. A distinct frequency maximum in the range of 50-70 residues was identified (Supplementary Fig. S6) and thus we introduced a length cutoff of 100 amino acids (proteins thus being below 12 kDa) for futher analysis. The term "small zinc finger protein" is used throughout the manuscript to refer to members of this class of proteins which are characterized by having at least one CPxCG-related zinc finger motif and which are shorter than 100 residues.

Identification of small zinc finger proteins in archaeal and bacterial genomes.

The analysis is based on 32 completely sequenced archaeal genomes (as illustrated in Supplementary Fig. S7, the underlying genomes are listed in Supplementary Table S3) and on 24 bacterial genomes. For genomes sequenced and annotated in our department, data were extracted from HaloLex (www.halolex.mpg.de) while the remainder of the genomes were retrieved from NCBI (www.ncbi.nlm.nih.gov) as made available through the MiGenAS system (Rampp *et al*., 2006). This provided the annotated protein-coding gene set. To allow detection of yet unannotated proteins we used six-frame translation data allowing ATG and GTG start codons and a minimum protein length of 30 amino acids. ORFs from sixframe translation were mapped to the annotated protein-coding gene set based on the position of the stop codon which is unambigous (while the position of the start codon differs in case of

alternative start codon selection). For a small subset, mapping of protein-coding genes to sixframes failed, e.g. for genes with a TTG start codon which was not considered upon sixframe translation. The number of protein-coding genes (total and mapped), of annotated and yet unannotated small zinc finger proteins and their relative abundance compared to the overall gene set (provided as % value) was computed (for archaea Supplementary Table S4, for bacteria Supplementary Table S5).

Figure legends

Supplementary Fig. S1: Initial processing scheme for bioinformatic analysis.

Analysis started with annotated protein-coding genes from 32 completely sequenced archaeal genomes. Proteins with at least 2 general Cys/His patterns were selected of which at least one was requested to be CPxCG-like. For distance analysis, the set was restricted to proteins having exactly 2 patterns. The set was subdivided into (a) proteins having 2 CPxCG-like patterns and (b) proteins having one CPxCG-like pattern and one general Cys/His pattern. This set was further subdivided to have the CPxCG-like pattern either first or second.

Supplementary Fig. S2: Distance analysis for pairs of CPxCG-like patterns.

The number of proteins having a given distance between the pair of CPxCG-like patterns is plotted (open circles, dotted line). The cumulative fraction of proteins having pattern pairs up to this distance is also shown (filled circles, contigous line). With one exception, no proteins with patterns closer than 7 residues are found, while 97% of the pattern pairs have a distance of up to 40 residues. Correspondingly, a distance cutoff of 7-40 residues (indicated by the black bar) was introduced in the motif detection algorithm. A total of 864 pattern pairs was analyzed.

Supplementary Fig. S3: Distance analysis for pairs of CPxCG-like and general Cys/His patterns.

The cumulative fraction of proteins having pattern pairs up to this distance is shown for three subsets. Data for proteins with two CPxCG-like patterns are redrawn from Supplementary Fig. S2 for comparison (filled circles) and the distance cutoff values are indicated by vertical lines. Data for pairs with one CPxCG-like pattern and one general Cys/His pattern are shown. In such pairs, the CPxCG-like pattern may be first (open squares) or second (filled triangles). While 97% of the proteins with two CPxCG-like patterns have a distance of up to 40 residues, this value is significantly lower for the less specific pattern pairs (CPxCG-like pattern first: 75% of 677 motif pairs, second: 69% of 513 motif pairs).

Supplementary Fig. S4: Frequency analysis of the general Cys/His pattern type.

The relative frequency for the 8 possible types has been computed for 1095 combinations of the general Cys/His pattern with a CPxCG-like pattern. Data were computed separately for motif pairs within the distance limits of 7-40 residues (a, CPxCG-related zinc finger motif) and for those with longer distances (b). A strong bias towards the CxxCx type is evident for motif pairs within distance limits. No such preference exists for long distance pairs.

Supplementary Fig. S5: Handling of overlapping and very closely spaced patterns.

Three principle configurations of overlapping or very closely spaced patterns are shown by sequence examples. In each case, alternative Cys/His patterns can be extracted as illustrated in the grey box above the sequence examples. The pattern selected by our algorithm is indicated by bold underlining and the basis of the decision is explained in a comment. **A**: this configuration in which the second Cys/His is unambigous, allows the extraction of a patterns with two or a pattern with three intermediate amino acids. As indicated, the pattern with two intermediate amino acids is extracted. **B**: The central Cys/His can be coupled either to the first

or to the third. As indicated, we prefer a CPxCG-like pattern over a general Cys/His pattern. When both solutions are equivalent we use the first pattern by default. **C**: When two patterns are very closely spaced, a CPxCG-like pattern triggers suppression of a general Cys/His pattern.

Supplementary Fig. S6: Protein length statistics.

This analysis is based on proteins annotated in 32 archaeal genomes that contain at least one CPxCG-related zinc finger motif, excluding proteins that contain Prosite motifs PS00198 or PS00202. The number of proteins within a length window of 10 amino acids (e.g. 10-19 for length 10) is indicated. Inset: the cake diagram shows that 23% of the proteins are shorter than 100 residues. A corresponding cutoff was implemented for further analysis (broken line).

Supplementary Fig. S7: Final processing scheme for bioinformatic analysis.

Using 32 completely sequenced archaeal genomes, the protein-coding genes were selected. Filtering of overlapping and closely spaced patterns is implemented. Pattern pairs having at least one CPxCG-like pattern and being within distance limits (7-40 residues) are considered to form a CPxCG-related zinc finger motif. Proteins matching to Prosite motifs PS00198 or PS00202 were eliminated. Of the 3165 proteins with at least one CPxCG-related zinc finger motif, 3101 can be mapped to the corresponding sixframe translations. Finally, proteins shorter than 100 residues are selected.

References

Gattiker, A., Gasteiger, E., Bairoch A., (2002) ScanProsite: a reference implementation of a PROSITE scanning tool. *Appl Bioinformatics*. 1(2):107-108.

Rampp, M., Soddemann, T., Lederer, H., (2006) The MIGenAS integrated bioinformatics toolkit for web-based sequence analysis *Nucleic Acids Res*. **34**:W15-9.

Saeed, A.I., Sharov, V., White, J., Li, J., Liang, W., Bhagabati, N., Braisted, J., Klapa, M., Currier, T., Thiagarajan, M., Sturn, A., Snuffin, M., Rezantsev, A., Popov, D., Ryltsov, A., Kostukovich, E., Borisovsky, I., Liu, Z., Vinsavich, A., Trush, V., Quackenbush, J., (2003) TM4: a free, open-source system for microarray data management and analysis. *Biotechniques* **34**: 374-378

Tusher, V.G., Tibshirani, R., Chu, G., (2001) Significance analysis of microarrays applied to the ionizing radiation response. *Proc Natl Acad Sci USA* **98**: 5116-5121

Supplementary Table S1

Regulation factors represent the relative intensity as computed from the log_2 ratio. Negative values indicate down-regulation while positive values indicate up-regulation. The term "regulation factor" is also used for the bop-related but non-regulated genes. Values in square brackets indicate data with a false discovery rate above 5% which is a consequence of the differences being minimal ("regulation" factors below 1.2).

Downregulated genes in the BrzC11S mutant

ID	regulation factor	gene	protein name
OE7065F	-17.3	cydA1	cytochrome d ubiquinol oxidase chain I
OE3095R	-7.2	-	hypothetical protein
OE6030R	-4.7	-	conserved hypothetical protein
OE3093R	-4.5	crtB1	phytoene synthase
OE3107F	-3.8	-	conserved hypothetical protein
OE4673F	-3.5	cxp	carboxypeptidase
OE4670F	-3.4	-	conserved hypothetical protein
OE2013R	-3.2	acd4	probable acyl/butyryl-CoA dehydrogenase
OE2808F	-2.8	-	hypothetical protein
OE3106F	-2.6	bop	bacteriorhodopsin precursor
OE6114R	-2.2	-	hypothetical protein
OE3688F	-2.1	-	hypothetical protein
OE2732R	-2.1	-	conserved hypothetical protein
OE1202F	-2.1	trp1	probable ABC-type transport system ATP-binding/permease protein
OE4563F	-2.0	-	hypothetical protein
OE3942R	-2.0	-	hypothetical protein
OE3468R	-2.0	crtI2	phytoene dehydrogenase

Upregulated genes in the BrzC11S mutant

ID	regulation factor	gene	protein name
OE5168F	16.5	ugpA	probable ABC-type transport system permease protein
OE5166F	9.9	ugpB	probable ABC-type transport system periplasmic substrate-binding protein
OE4398F	9.6	hal	O-acetylhomoserine (thiol)-lyase
OE4479R	9.2	pstB1	probable ABC-type phosphate transport system ATP-binding protein
OE4485R	7.6	phoX1	probable ABC-type phosphate transport system periplasmic substrate-binding protein
OE4612F	7.2	hly	halolysin R4
OE6098R	6.3	-	conserved hypothetical protein
OE5149R	6.1	-	hypothetical protein
OE2854R	6	-	hypothetical protein
OE2100R	5.8	spoVR	spore cortex formation protein homolog

**OE3766R**	**5.7**	**-**	**hypothetical protein**
**OE2108F**	**5**	**-**	**conserved hypothetical protein**
**OE3526R**	**4.7**	**-**	**nuclease homolog**
**OE3098R**	**4**	**aaa2**	**AAA-type ATPase (transitional ATPase homolog)**
**OE1775R**	**3.8**	**-**	**hypothetical protein**
**OE3960F**	**3.8**	**nhaC2**	**Na+/H+-exchanging protein**
**OE4399F**	**3.7**	**oxr8**	**probable oxidoreductase (glycerol-3-phosphate dehydrogenase homolog)**
**OE6157R**	**3.5**	**-**	**hypothetical protein**
**OE1774R**	**3.4**	**-**	**hypothetical protein**
**OE5192R**	**3.3**	**aph**	**alkaline phosphatase**
**OE4480R**	**3.2**	**pstA1**	**probable ABC-type phosphate transport system permease protein**
**OE1447R**	**3.2**	**-**	**conserved hypothetical protein**
**OE2442R**	**3.1**	**-**	**hypothetical protein**
**OE7033R**	**3.1**	**gvpD1**	**gas-vesicle operon protein gvpD1 (probable repressor protein)**
**OE4483R**	**3.1**	**pstC1**	**probable ABC-type phosphate transport system permease protein**
**OE6074R**	**3**	**-**	**hypothetical protein**
**OE1448R**	**3**	**-**	**conserved hypothetical protein**
**OE3744R**	**3**	**-**	**hypothetical protein**
**OE3936F**	**3**	**pchB**	**potassium channel protein homolog**
**OE5090F**	**2.9**	**-**	**hypothetical protein**
**OE6097R**	**2.9**	**-**	**conserved hypothetical protein**
**OE2378R**	**2.8**	**parA1**	**parA domain protein**
**OE7030R**	**2.8**	**gvpG1**	**gas-vesicle operon protein gvpG1**
**OE7032R**	**2.7**	**gvpE1**	**gas-vesicle operon protein gvpE1 (probable activator protein)**
**OE2201F**	**2.7**	**chiA1**	**chitinase**
**OE6093F**	**2.6**	**phzF**	**probable phenazine biosynthesis protein**
**OE5160F**	**2.6**	**gldA1**	**glycerol dehydrogenase**
**OE1232R**	**2.6**	**-**	**conserved hypothetical protein (nonfunctional, N-terminal part, interrupted by ISH2)**
**OE1789R**	**2.5**	**-**	**conserved hypothetical protein**
**OE5370R**	**2.5**	**-**	**hypothetical protein**
**OE1584R**	**2.5**	**-**	**conserved hypothetical protein**
**OE3509R**	**2.4**	**-**	**conserved hypothetical protein**
**OE3341F**	**2.4**	**-**	**hypothetical protein**
**OE6096A1R**	**2.3**	**-**	**hypothetical protein**
**OE7024R**	**2.3**	**gvpK1**	**gas-vesicle operon protein gvpK1**
**OE5124R**	**2.3**	**gvpD2**	**gas-vesicle operon protein gvpD2 (probable repressor protein)**
**OE3741R**	**2.3**	**-**	**hypothetical protein**
**OE4244F**	**2.2**	**-**	**conserved hypothetical protein**
**OE2117F**	**2.2**	**-**	**conserved hypothetical protein**
**OE2206F**	**2.2**	**chiA3**	**probable chitinase**
**OE2103R**	**2.2**	**prkA2**	**protein kinase prkA homolog**
**OE1495R**	**2.2**	**pykA**	**pyruvate kinase**
**OE2104R**	**2.2**	**prkA1**	**probable protein kinase prkA (serine kinase)**
**OE1934R**	**2.1**	**edp**	**proteinase IV (EC 3.4.-.-)**
**OE5208R**	**2.1**	**arcA**	**arginine deiminase**
**OE5170F**	**2.1**	**ugpC**	**probable ABC-type transport system ATP-binding protein**
**OE7031R**	**2**	**gvpF1**	**gas-vesicle operon protein gvpF1**
**OE4688F**	**2**	**-**	**conserved hypothetical protein**
**OE1952F**	**2**	**purE**	**phosphoribosylaminoimidazole carboxylase; catalytic chain**
**OE1005F**	**2**	**-**	**conserved hypothetical protein**
**OE3947R**	**2**	**-**	**conserved hypothetical protein**
**OE5102R**	**2**	**-**	**IS1341-type transposase (ISH12)**
**OE1641R**	**2**	**fad2**	**3-hydroxybutryl-CoA dehydratase**

Non-regulated *bop*-related genes in the BrzC11S mutant

ID	regulation factor	gene	protein name
OE3102R	1.1	brp	bacteriorhodopsin-related protein
OE3100F	-1.6	blp	bacterioopsin-linked protein blp
OE3101R	1.1	bat	bacterioopsin activator
OE2448F	1.5	boa4	homolog to transcription regulator bat
OE3134F	1.1	boa2	homolog to transcription regulator bat
OE3980R	-1.2	blh	brp-like protein

Downregulated genes in the BrzH52F mutant

ID	regulation factor	gene	protein name
OE7065F	-12.4	cydA1	cytochrome d ubiquinol oxidase chain I
OE3095R	-6.0	-	hypothetical protein
OE6030R	-4.7	-	conserved hypothetical protein
OE3093R	-2.9	crtB1	phytoene synthase
OE2732R	-2.8	-	conserved hypothetical protein
OE3107F	-2.7	-	conserved hypothetical protein
OE3042F	-2.2	-	hypothetical protein
OE3666F	-2.0	-	conserved hypothetical protein
OE3100F	-2.0	blp	bacterioopsin-linked protein blp
OE3106F	-2.0	bop	bacteriorhodopsin precursor
OE2012R	-2.0	-	hypothetical protein

Upregulated genes in the BrzH52F mutant

ID	regulation factor	gene	protein name
OE6098R	6.6	-	conserved hypothetical protein
OE2100R	5.8	spoVR	spore cortex formation protein homolog
OE4313F	3.6	appB	ABC-type transport system permease protein
OE3960F	3.5	nhaC2	Na+/H+-exchanging protein
OE4311F	3.3	appA	ABC-type transport system periplasmic substrate-binding protein
OE6097R	3.1	-	conserved hypothetical protein
OE6074R	2.9	-	hypothetical protein
OE4398F	2.8	hal	O-acetylhomoserine (thiol)-lyase
OE3116F	2.8	-	conserved hypothetical protein
OE2442R	2.6	-	hypothetical protein
OE6096A1R	2.3	-	hypothetical protein
OE4316F	2.3	appC	ABC-type transport system permease protein
OE5370R	2.3	-	hypothetical protein
OE3509R	2.3	-	conserved hypothetical protein
OE1232R	2.2	-	conserved hypothetical protein (nonfunctional
OE7032R	2.1	gvpE1	gas-vesicle operon protein gvpE1

Non-regulated *bop*-related genes in the BrzH52F mutant

ID	regulation factor	gene	protein name
OE3102R	1.4	brp	bacteriorhodopsin-related protein
OE3101R	-1.1	bat	bacterioopsin activator
OE2448F	[1.0]	boa4	homolog to transcription regulator bat
OE3134F	[1.1]	boa2	homolog to transcription regulator bat

OE3980R	[-1.0]	blh	brp-like protein

Downregulated genes in the stopBrz mutant

ID	regulation factor	gene	protein name
OE3095R	-5.4	-	hypothetical protein
OE4673F	-3.9	cxp	carboxypeptidase
OE3093R	-3.6	crtB1	phytoene synthase
OE3106F	-2.8	bop	bacteriorhodopsin precursor
OE3107F	-2.7	-	conserved hypothetical protein
OE2013R	-2.6	acd4	probable acyl/butyryl-CoA dehydrogenase
OE2808F	-2.6	-	hypothetical protein
OE7093R	-2.1	idi1a	isopentenyl-diphosphate delta-isomerase
OE4759F	-2.0	csg	cell surface glycoprotein precursor
OE3807R	-2.0	yjbG	oligoendopeptidase

Upregulated genes in the stopBRZ mutant

ID	regulations factor	gene	protein name
OE5166F	9.9	ugpB	probable ABC-type transport system periplasmic substrate-binding protein
OE4479R	9.2	pstB1	probable ABC-type phosphate transport system ATP-binding protein
OE4485R	5.6	phoX1	probable ABC-type phosphate transport system periplasmic substrate-binding protein
OE7034F	4.9	-	gas-vesicle protein gvpA1
OE3098R	4.5	aaa2	AAA-type ATPase (transitional ATPase homolog)
OE4398F	4.4	hal	O-acetylhomoserine (thiol)-lyase
OE6098R	4.2	-	conserved hypothetical protein
OE2100R	4.2	spoVR	spore cortex formation protein homolog
OE2854R	4.1	-	hypothetical protein
OE3766R	3.7	-	hypothetical protein
OE5125F	3.5	gvpA2	gas-vesicle protein gvpA2
OE3960F	3.4	nhaC2	Na+/H+-exchanging protein
OE4480R	3.4	pstA1	probable ABC-type phosphate transport system permease protein
OE3526R	3.3	-	nuclease homolog
OE3936F	3.3	pchB	potassium channel protein homolog
OE5168F	3.3	ugpA	probable ABC-type transport system permease protein
OE1775R	3.1	-	hypothetical protein
OE5192R	2.8	aph	alkaline phosphatase
OE5160F	2.8	gldA1	glycerol dehydrogenase
OE1774R	2.7	-	hypothetical protein
OE2442R	2.5	-	hypothetical protein
OE4399F	2.5	oxr8	probable oxidoreductase
OE4483R	2.5	pstC1	probable ABC-type phosphate transport system permease protein
OE1767F	2.4	-	conserved hypothetical protein
OE1934R	2.3	edp	proteinase IV
OE5127F	2.3	gvpC2	gas-vesicle protein gvpC2
OE4688F	2.2	-	conserved hypothetical protein
OE1641R	2.2	fad2	3-hydroxybutryl-CoA dehydratase
OE4244F	2.2	-	conserved hypothetical protein
OE4612F	2.1	hly	halolysin R4
OE1952F	2.1	purE	phosphoribosylaminoimidazole carboxylase catalytic chain
OE1584R	2.1	-	conserved hypothetical protein

**OE1448R**	**2.1**	**-**	**conserved hypothetical protein**
**OE4449R**	**2.1**	**-**	**hypothetical protein**
**OE2201F**	**2.1**	**chiA1**	**chitinase**
**OE4505F**	**2.0**	**pheS**	**phenylalanine--tRNA ligase alpha chain**
**OE3617F**	**2.0**	**-**	**conserved hypothetical protein**
**OE1495R**	**2.0**	**pykA**	**pyruvate kinase**
**OE2749F**	**2.0**	**urk**	**uridine kinase**
**OE1675R**	**2.0**	**pstB2**	**probable ABC-type phosphate transport system ATP-binding protein**

Non-regulated *bop*-related genes in the stopBRZ mutant

ID	regulation factor	gene	protein name
**OE3102R**	**[-1.1]**	**brp**	**bacteriorhodopsin-related protein**
**OE3100F**	**-1.7**	**blp**	**bacterioopsin-linked protein blp**
**OE3101R**	**[-1.2]**	**bat**	**bacterioopsin activator**
**OE2448F**	**1.5**	**boa4**	**homolog to transcription regulator bat**
**OE3134F**	**1.1**	**boa2**	**homolog to transcription regulator bat**
**OE3980R**	**-1.1**	**blh**	**brp-like protein**

Supplementary Table S2 Primers used in this work

Name of primer	Sequences
fp1	5’-AATCGAAAGCTTGACCGAGCTGAACGCGTAGA-3’
rp1	5’-ACGAGCATCTAGAGGTCTAACAACAACAGC-3’
fp2	5’-GACGTGAAGATGGGGCTCCCGTAAGGTGCAACCGTGAAG-3’
rp2	5’-CGGACTTCACGGTTGCACCTTACGGGAGCCCCATCTTCA-3’
fp3	5’-CTCCACTGTCCGCGGTCCGGATCCGACGTGAAG-3’
rp3	5’-CTTCACGTCGGATCCGGACCGCGGACAGTGGAG-3’
fp4	5’-TGAGTGCCGCAACGATTTCGAGTTTTTCGTGCGC-3’
rp4	5’-GCGCACGAAAAACTCGAAATCGTTGCGGCACTCA-3’
fp5	5’-TCGTGCGCCTGCAGTGGTAACACGCGTG-3’
rp5	5’-CTACTCCCTGCAGTGTCTTCCCGTTCCTT-3’
fp6	5’-GACGCTCGTCCCAGCCATCG -3’
rp6	5’-AGCCGACCTTCGCGCTCACG -3’
fp7	5’-CCGACCGCATCGAGCAGAGC -3’
rp7	5’-CGCGGTCCAGCAGTCGATCC -3’

Supplementary Table S3: Analyzed archaeal genomes

The table lists the organism (full name and abbreviation), the accession and length of the longest contig (assuming this to be the major chromosome), the number of additional contigs (e.g. plasmids), the overall genome length and the number of annotated genes. Data are taken from NCBI except for those marked.

[a]: The data for these organisms was taken from HaloLex (www.halolex.mpg.de)

Organism	Full species name	Genome length	Accession of longest contig	Length of longest contig	Number of additional contigs	Total number of annotated genes
Aful	Archaeoglobus fulgidus	2178400	NC_000917	2178400	0	2420
Aper	Aeropyrum pernix	1669696	NC_000854	1669696	0	1700
Hasal [a]	Halobacterium salinarum	2668776	-	2000962	4	2886
Hmar	Haloarcula marismortui	4274642	NC_006396	3131724	8	4240
Hqwal [a]	Haloquadratum walsbyi	3179361	NC_008212	3132494	1	2920
Hsal	Halobacterium sp.NRC-1	2571010	NC_002607	2014239	2	2622
Mace	Methanosarcina acetivorans	5751492	NC_003552	5751492	0	4540
Metba	Methanosarcina barkeri	4873766	NC_007355	4837408	1	3624
Metbu	Methanococcoides burtonii	2575032	NC_007955	2575032	0	2273
Methe	Methanosaeta thermophila	1879471	NC_008553	1879471	0	1696
Methu	Methanospirillum hungatei	3544738	NC_007796	3544738	0	3139
Metja	Methanococcus jannaschii	1739927	NC_000909	1664970	2	1786
Metka	Methanopyrus kandleri	1694969	NC_003551	1694969	0	1687
Metst	Methanosphaera stadtmanae	1767403	NC_007681	1767403	0	1534
Metth	Methanobacterium thermoautotrophicum	1751377	NC_000916	1751377	0	1873
Mmar	Methanococcus maripaludis	1661137	NC_005791	1661137	0	1722
Mmaz	Methanosarcina mazei	4096345	NC_003901	4096345	0	3370
Napha [a]	Natronomonas pharaonis	2749696	NC_007426	2595221	2	2862
Nequi	Nanoarchaeum equitans	490885	NC_005213	490885	0	536
Ptor	Picrophilus torridus	1545895	NC_005877	1545895	0	1535
Pyrab	Pyrococcus abyssi	1768562	NC_000868	1765118	1	1898
Pyrae	Pyrobaculum aerophilum	2222430	NC_003364	2222430	0	2605
Pyrfu	Pyrococcus furiosus	1908256	NC_003413	1908256	0	2125
Pyrho	Pyrococcus horikoshii	1738505	NC_000961	1738505	0	1955
Pyris	Pyrobaculum islandicum	1826402	NC_008701	1826402	0	1978
Ssol	Sulfolobus solfataricus	2992245	NC_002754	2992245	0	2977
Stok	Sulfolobus tokodaii	2694756	NC_003106	2694756	0	2825
Sulac	Sulfolobus acidocaldarius	2225959	NC_007181	2225959	0	2223
Taci	Thermoplasma acidophilum	1564906	NC_002578	1564906	0	1482
Theko	Thermococcus kodakaraensis	2088737	NC_006624	2088737	0	2306
Thepe	Thermofilum pendens	1813393	NC_008698	1781889	1	1876
Tvol	Thermoplasma volcanium	1584804	NC_002689	1584804	0	1499

Supplementary Table S4: CPxCG-related zinc finger proteins in 32 archaeal genomes

For 32 archaeal genomes, proteins with less than 100 residues were counted. Annotated proteins are those found in the NCBI version of the genome (except for Hasal, Hqwal, and Napha). ZnF proteins refer to proteins having at least one CPxCG-related zinc finger motif according to the algorithm described in Materials and Methods. ZnF proteins are counted in the annotated gene set and for additional unmapped sixframe translations. Sixframe translations require an ATG or GTG start codon and a minimum ORF length of 30 residues. The full organism names are shown in Table S3.

			small ZnF proteins						
**Organism**	**annotated proteins**	**small proteins (<100aa)**	**annotated proteins**	**% annotated of small proteins**	**non-annotated sixframes**	**% non-annotated of small proteins**	**total number of proteins**	**% total of small proteins**	**% non-annotated of total znf**
Aful	2420	318	21	6.6	18	5.7	39	12.3	46.2
Aper	1700	181	16	8.8	7	3.9	23	12.7	30.4
Hasal	2886	477	58	12.2	11	2.3	69	14.5	15.9
Hmar	4240	625	56	9.0	59	9.4	115	18.4	51.3
Hqwal	2920	461	35	7.6	51	11.1	86	18.7	59.3
Hsal	2622	386	36	9.3	31	8.0	67	17.4	46.3
Mace	4540	569	21	3.7	49	8.6	70	12.3	70.0
Metba	3624	404	32	7.9	43	10.6	75	18.6	57.3
Metbu	2273	218	21	9.6	31	14.2	52	23.9	59.6
Methe	1696	161	20	12.4	46	28.6	66	41.0	69.7
Methu	3139	297	25	8.4	41	13.8	66	22.2	62.1
Metja	1786	183	18	9.8	6	3.3	24	13.1	25.0
Metka	1687	136	15	11.0	3	2.2	18	13.2	16.7
Metst	1534	155	18	11.6	3	1.9	21	13.5	14.3
Metth	1873	230	19	8.3	16	7.0	35	15.2	45.7
Mmar	1722	174	16	9.2	8	4.6	24	13.8	33.3
Mmaz	3370	382	22	5.8	51	13.4	73	19.1	69.9
Napha	2862	397	45	11.3	19	4.8	64	16.1	29.7
Nequi	536	49	7	14.3	7	14.3	14	28.6	50.0
Ptor	1535	133	10	7.5	16	12.0	26	19.5	61.5
Pyrab	1898	195	16	8.2	1	0.5	17	8.7	5.9
Pyrae	2605	451	22	4.9	16	3.5	38	8.4	42.1
Pyrfu	2125	278	15	5.4	3	1.1	18	6.5	16.7
Pyrho	1955	213	15	7.0	2	0.9	17	8.0	11.8
Pyris	1978	240	22	9.2	25	10.4	47	19.6	53.2
Ssol	2977	236	15	6.4	15	6.4	30	12.7	50.0
Stok	2825	268	10	3.7	11	4.1	21	7.8	52.4
Sulac	2223	230	19	8.3	7	3.0	26	11.3	26.9
Taci	1482	110	13	11.8	16	14.5	29	26.4	55.2
Theko	2306	300	15	5.0	9	3.0	24	8.0	37.5

Thepe	1876	196	17	8.7	6	3.1	23	11.7	26.1
Tvol	1499	134	12	9.0	5	3.7	17	12.7	29.4
**mean**	**2334.8**	**274.6**	**21.9**	**8.0**	**19.8**	**7.2**	**41.7**	**15.2**	**47.5**

Supplementary Table S5: CPxCG-related zinc finger proteins in 24 bacterial genomes

The structure of the table corresponds to Table S4.

			small ZnF proteins						
**Organism**	**annotated proteins**	**small proteins (<100aa)**	**anno**	**% anno of small proteins**	**non-anno sixframes**	**% non-anno of small proteins**	**total**	**% total of small proteins**	**% non-anno of total znf**
Anava	5661	599	9	1.5	27	4.5	36	6.0	75.0
Aquae	1560	42	2	4.8	6	14.3	8	19.0	75.0
Bbac	3587	255	5	2.0	46	18.0	51	20.0	90.2
Bsubt	4105	476	8	1.7	35	7.4	43	9.0	81.4
Caucr	3737	284	5	1.8	7	2.5	12	4.2	58.3
Chltr	895	62	2	3.2	3	4.8	5	8.1	60.0
Dehet	1580	284	14	4.9	4	1.4	18	6.3	22.2
Ecoli	4131	380	12	3.2	47	12.4	59	15.5	79.7
Fnuc	2067	218	4	1.8	1	0.5	5	2.3	20.0
Hpyl	1489	117	1	0.9	1	0.9	2	1.7	50.0
Neigo	2002	330	3	0.9	42	12.7	45	13.6	93.3
Pgin	1909	229	1	0.4	23	10.0	24	10.5	95.8
Pirsp	7325	1931	11	0.6	98	5.1	109	5.6	89.9
Pmar	1717	266	0	0.0	3	1.1	3	1.1	100.0
Psefl	5736	399	6	1.5	110	27.6	116	29.1	94.8
Rhosh	4242	307	3	1.0	30	9.8	33	10.7	90.9
SPCC	3569	359	0	0.0	22	6.1	22	6.1	100.0
Salru	2833	177	20	11.3	22	12.4	42	23.7	52.4
Scoe	8154	630	16	2.5	98	15.6	114	18.1	86.0
Spne	2043	317	1	0.3	3	0.9	4	1.3	75.0
Tmar	1858	163	5	3.1	6	3.7	11	6.7	54.5
Tpal	1036	117	4	3.4	37	31.6	41	35.0	90.2
Tthe	2238	197	5	2.5	11	5.6	16	8.1	68.8
Vcho	3835	597	7	1.2	39	6.5	46	7.7	84.8
**mean**	**3221.2**	**364.0**	**6.0**	**1.5**	**30.0**	**8.2**	**36.0**	**9.9**	**83.3**

Fig.S1

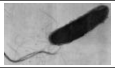	32	archaeal genomes
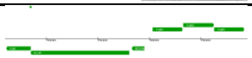	74714	annotated proteins
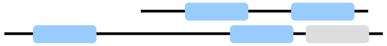	4475	proteins with 2 or more patterns
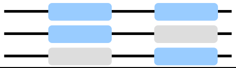	2054	proteins with exactly 2 patterns
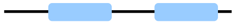	864	proteins with 2 CPxCG-like patterns
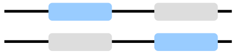	1190	proteins with 1 CPxCG-like pattern and one more general Cys/His pattern
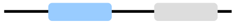	677	proteins with CPxCG-like before Cys/His
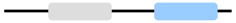	513	proteins with Cys/His before CPxCG-like

Fig.S2

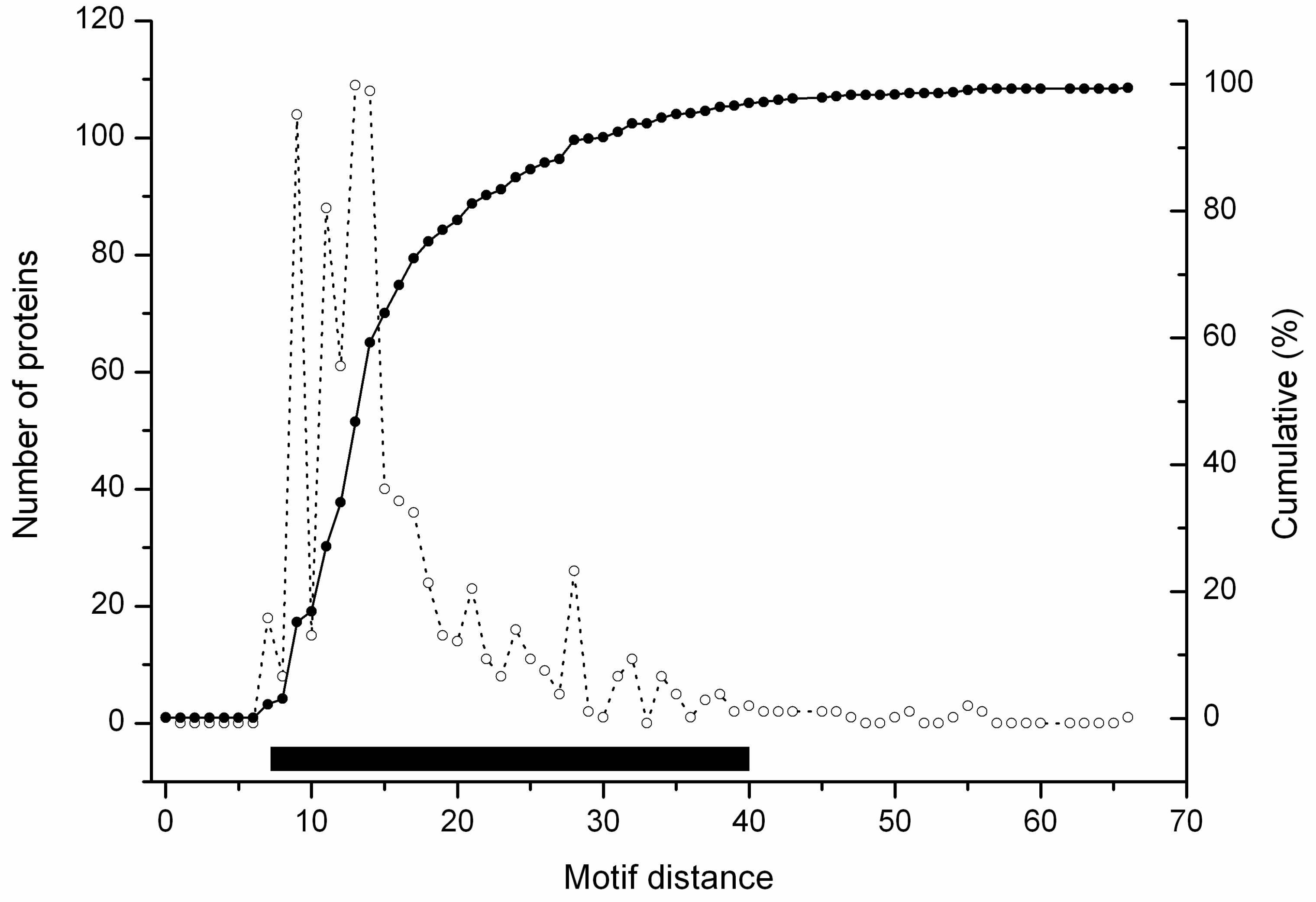

120
100
80
60
40
20
0
Number of proteins
100
80
60
40
20
0
Cumulative (%)
0
10
20
30
40
50
60
70
Motif distance

Fig.S3

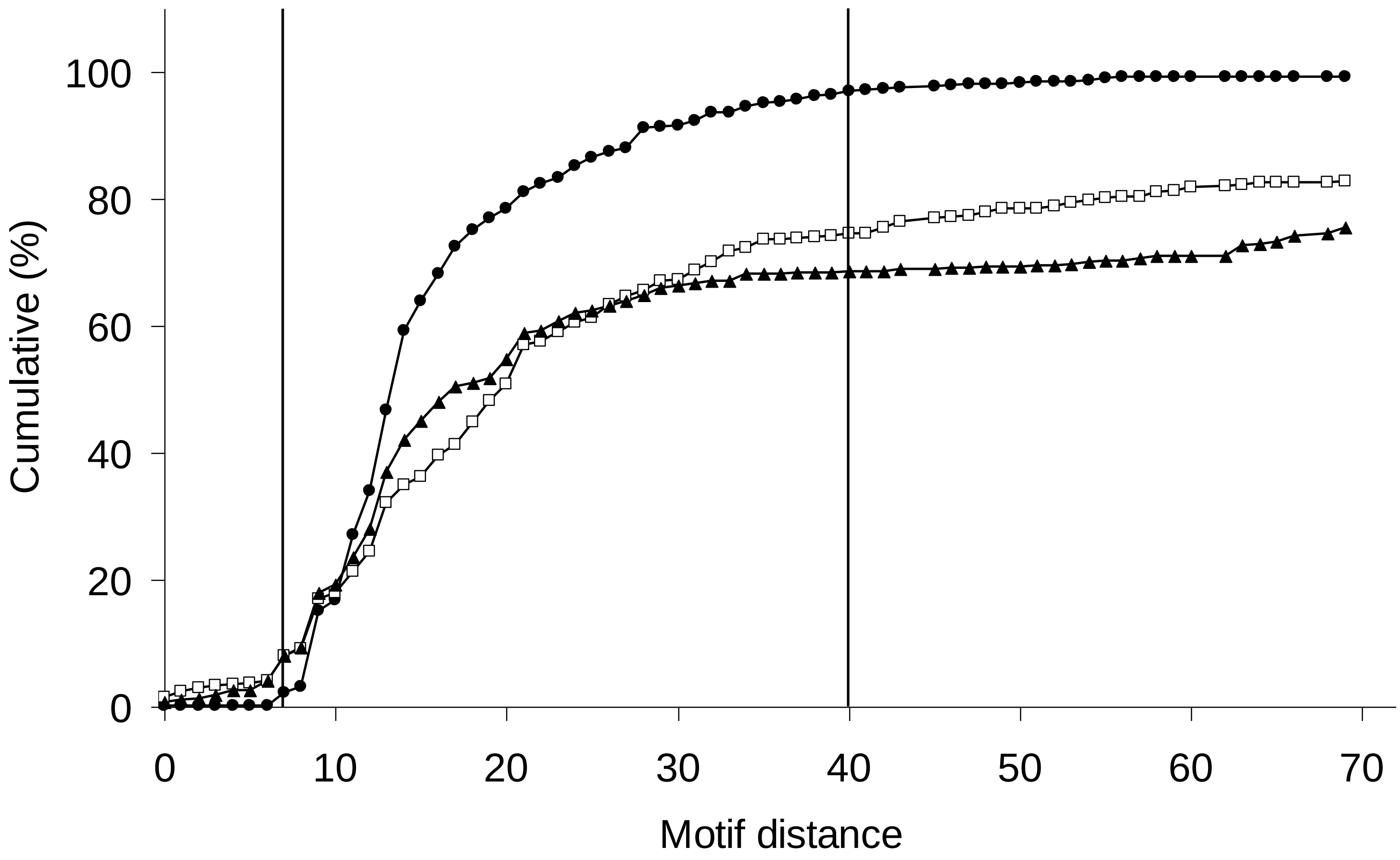

Cumulative (%)
0
20
40
60
80
100
0
10
20
30
40
50
60
70
Motif distance

Fig.S4

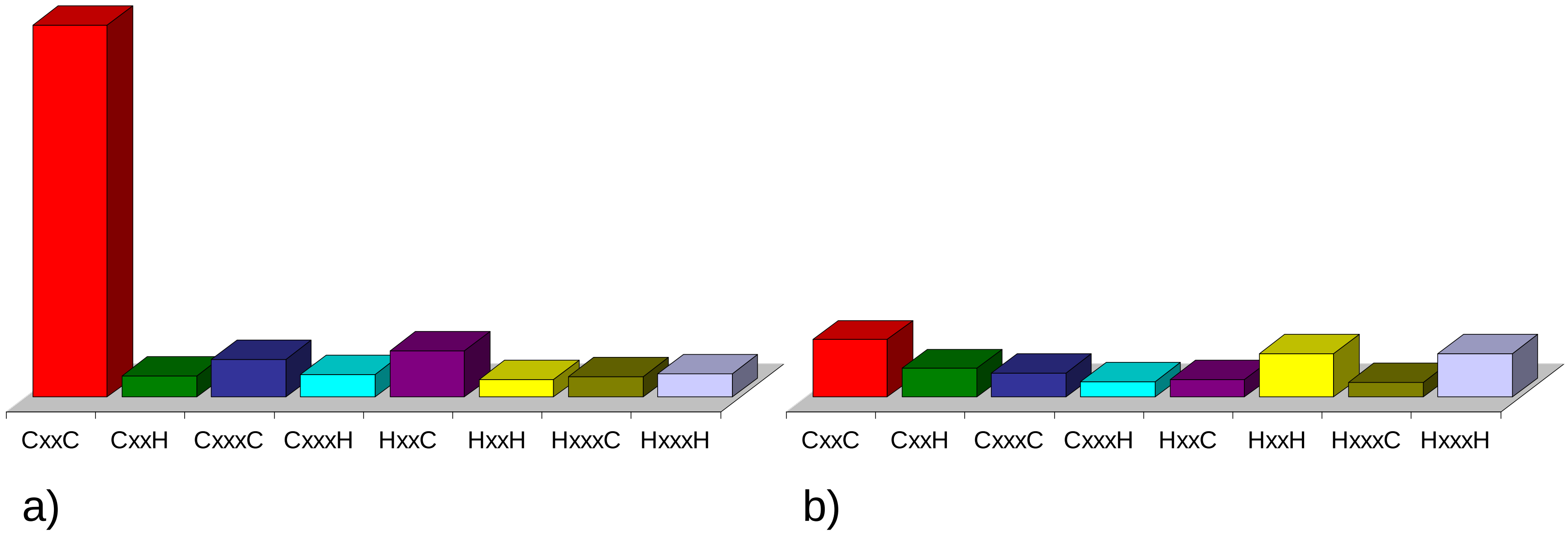
CxxC
CxxH
CxxxC
CxxxH
HxxC
HxxH
HxxxC
HxxxH
a)
CxxC
CxxH
CxxxC
CxxxH
HxxC
HxxH
HxxxC
HxxxH
b)

Fig.S5

**A**	*handling of CCxxCx*
CxxxCx<br>CxxCx	longer potential pattern<br>shorter potential pattern
C**<u>CAxCA</u>**	the pattern with two intermediate residues is preferred over the pattern with three intermediate residues
**B**	*overlapping patterns*
CxxCx<br>CxxCx	first potential pattern<br>second potential pattern
**<u>CPxCP</u>**xCA<br>**<u>CPxCA</u>**xCA<br>CAx**<u>CPxCA</u>**<br>**<u>CAxCA</u>**xCA	both sub-patterns are CPxCG-like, first pattern is used<br>first sub-pattern is CPxCG-like, CPxCG-like pattern is used<br>second sub-pattern is CPxCG-like, CPxCG-like pattern is used<br>none of the sub-patterns is CPxCG-like, first pattern is used
**C**	*very closely spaced patterns*
CxxCx CxxCx	two very closely spaced patterns
**<u>CPxCG</u>**xxx**<u>CPxCG</u>**<br>**<u>CPxCG</u>**xxxCAxCA<br>CAxCAxxx**<u>CPxCG</u>**<br>**<u>CAxCA</u>**xxx**<u>CAxCA</u>**	both patterns are CPxCG-like, both are used<br>first pattern is CPxCG-like, CPxCG-like pattern is used<br>second pattern is CPxCG-like, CPxCG-like pattern is used<br>none of the patterns is CPxCG-like, both are used

Fig.S6

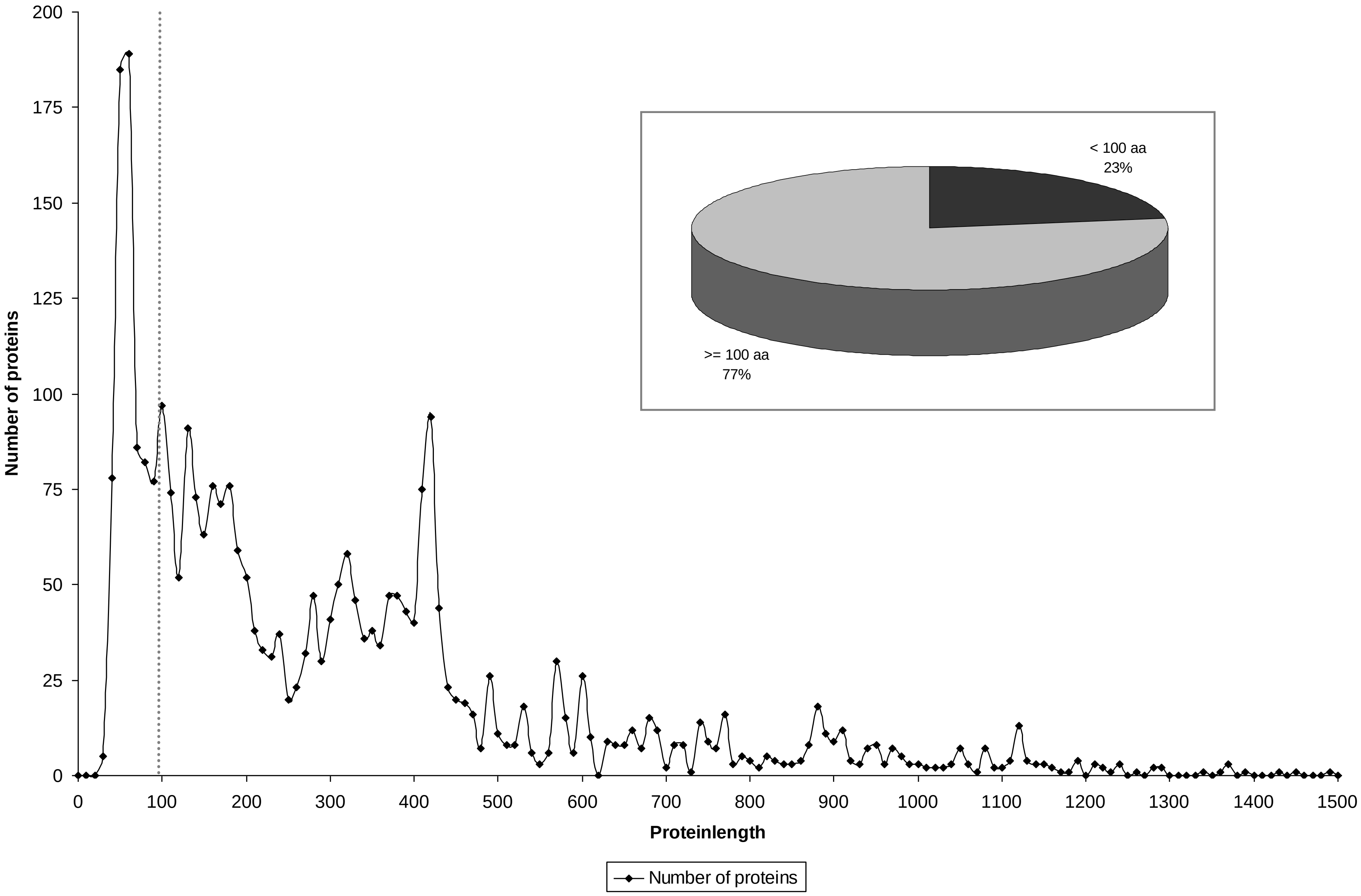

200
175
150
125
100
75
50
25
0
Number of proteins
0
100
200
300
400
500
600
700
800
900
1000
1100
1200
1300
1400
1500
Proteinlength
Number of proteins
< 100 aa
23%
>= 100 aa
77%

Fig.S7

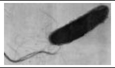	32	archaeal genomes
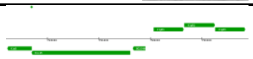	74665	proteins
Cxx**CPxCG**		resolve overlapping/closely spaced patterns
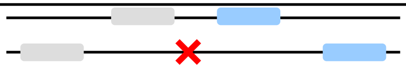		pair patterns into zinc finger motifs
~~PS00198~~ ~~PS00202~~	570	exclude ferredoxins / rubredoxins
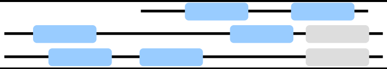	3165	proteins with at least 1 zinc finger motif
ATG…… ✓ GTG…… ✓ TTG…… ✗	3101	proteins that can be mapped to sixframes
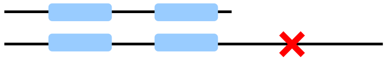	702	proteins shorter than 100 aa
